# The Hepaticojejunostomy Technique with Intra-Anastomotic Stent in Biliary Diseases and Its Evolution throughout the Years: A Technical Analysis

**DOI:** 10.1155/2016/3692096

**Published:** 2016-04-13

**Authors:** Demetrios Moris, Alexandros Papalampros, Michail Vailas, Athanasios Petrou, Michael Kontos, Evangelos Felekouras

**Affiliations:** 1st Department of Surgery, University of Athens Medical School, Laikon General Hospital, Agiou Thoma 17, 11527 Athens, Greece

## Abstract

Roux-en-Y hepaticojejunostomy (RYHJ) is currently considered as the definitive treatment for iatrogenic bile duct injuries and the principal representative of biliary diversion procedures. This technique has met many milestones of extensive evolution, particularly the last years of concomitant technological evolution (laparoscopic/robotic approach). Anastomotic strictures and leaks, which may have deleterious effects on the survival and quality of life of a patient with biliary obstruction of any cause, made the need of the development of a safe and efficient RYHJ compulsory. The aim of this technical analysis and the juxtaposed discussions is to elucidate with the most important milestones and technical tips and tricks all aspects of a feasible and reliable RYHJ technique that is performed in our center for the last 25 years in around 400 patients.

## 1. Introduction

Roux-en-Y hepaticojejunostomy (RYHJ) is currently considered as the definitive treatment for iatrogenic bile duct injuries [1]. It is a common operation, not only to bypass extrahepatic biliary obstructions, but also to establish biliary-enteric continuity after resections for benign and malignant diseases. Studies have shown good medium- and long-term outcomes following this procedure [1, 2]. Postoperative stricture formation at the anastomotic site varies throughout the literature from 4 to 38% of patients [3–5]. Untreated HJ stricture could lead to long-term complications such as choledocholithisis, cholangitis, liver abscess formation, secondary biliary cirrhosis, and portal hypertension [6]. Although revision HJ is required in about 20–25% of patients [7], the majority of such strictures can be treated by dilation via transhepatic or jejunal routes [8]. Indisputable tenets of this procedure include the creation of a durable jejunojejunostomy, followed by the creation of a tension-free anastomosis between the hepatic duct and the defunctionalized jejunal limb.

Anastomotic site stricture is a recognized complication of HJ. Bismuth-Corlette classification type of bile duct injury, revision surgery, nondilated proximal biliary system, and electrocautery damage are implicated in its occurrence [8].

The presence of dilated proximal bile duct is of paramount technical and clinical importance since when the ducts are dilated due to biliary obstruction, the anastomosis could be easy to constitute, which in turn minimizes the risk for postoperative complications but this is not the case in nondilated ducts.

It is a matter of debate among surgeons which operative technique must be chosen in order to prevent the anastomotic failures in cases with small nondilated ducts and whether the selective use of a transanastomotic stent could be of benefit in order to minimize the risk of stricture formation [9].

The aim of this technical analysis and the juxtaposed discussions is to elucidate with the most important milestones and technical tips and tricks all aspects of a feasible and reliable RYHJ technique with intra-anastomotic stenting with low leakage and stricture rates that can be successfully applied in a variety of biliary diseases; it is to be hoped that some global insights will emerge.

## 2. Milestones of the Evolution of Biliary Diversion Procedures

The history of biliary diversion procedures began almost a century ago, with the first report of choledochojejunostomy (CJ), the predecessor of hepaticojejunostomy (HJ), made in 1921 by Reid [10] whereas Maingot [11] presented the first case of concomitant cholecystectomy and CJ. The first report with the term hepaticojejunostomy (HJ) was made in the literature in 1949 by Sanders in a case of hemihepatectomy with HJ for irreparable defects of the bile ducts [12]. In 1950, Best introduced the use of T-tube in cases of CJ [13]. In 1952, Corff et al. [14] published the very first series of CJ with cholangiography whereas Allbritten Jr. introduced for the first time the term Roux-en-Y CJ (RYCJ) [15]. 1956 was a year of updates for CJ since 2 novel techniques of CJ were published, the Allen technique [16] and the Warren modification [17].

It was late 70s when the first evaluation of the feasibility and safety of RYHJ in the treatment of benign biliary diseases was published by Bismuth et al. [18] in a retrospective analysis of 123 patients. It was shown that this operation has 0% mortality rate and low learning curve and morbidity rate. The same year, Daugherty et al. [19] announced proximal hepatic duct reconstruction in benign and malignant biliary diseases using sutureless mucosal graft HJ, with all patients presenting with improvement of their symptoms postoperatively. A year later, the experience from Japan on intrahepatic pigment calculi treated with modified wraparound end-to-end HJ was presented to provide an effective and alternative method of treatment [20].

In 1984, Barker and Winkler [21] described a new technique of RYHJ with permanent access by involving the incorporation of a cutaneous access stoma in the Roux-en-Y loop of jejunum used for the anastomosis. This stoma provides permanent access to the anastomosis and to the hepatobiliary tree for nonoperative management of chronic and recurrent biliary tract problems.

In 1987, Bismuth et al. [22] announced the first application of RYHJ in the liver transplant setting as a safe and feasible approach to perform biliary anastomosis. In early 90s, there were the first data of the hedge-up comparison between RYHJ and jejunal interposition hepaticoduodenostomy to treat congenital dilation biliary tract diseases and the former was found superior in terms of postoperative reflux gastritis [23]. At the same period, Quintero et al. [24] published their data on RYHJ with subcutaneous access and the use of Gianturco stents as a method to control recurrent biliary strictures.

In 1998, the first experience of laparoscopic technique RYHJ in experimental setting with the application of transient endoluminally stented anastomosis (TESA) was announced [25]. This approach gave birth to the evolution of intra-anastomotic stenting and the laparoscopic approach when performing RYHJ.

At the end of the previous century, 2 technical advances of RYHJ were published. The first was a new technique of Hepp-Couinaud HJ using the posterior approach to the hepatic hilum, approach that was proven safe and feasible despite being evaluated as a case report [26], and the second was the first case controlled study evaluating the role and efficacy of laparoscopic RYHJ as a palliative treatment in the clinical setting of pancreatic cancer [27]. The results were encouraging in terms of mortality, morbidity, and length of hospitalization. In all categories, the laparoscopic approach was found superior compared to open RYHJ.

In 2002, Nagino et al. [28] developed new placement of RY jejunal limb in which the limb is placed via the retrocolic-retrogastric route in 133 consecutive obese patients and achieved tension-free anastomosis in all patients with neither early nor late complications directly related to this new reconstruction route occurring.

In 2004, the first robotically assisted laparoscopic RYHJ was performed in experimental setting and a feasibility study between the latter and pure laparoscopic and open approach took place [29]. The procedure was found feasible and safe but more time-consuming than the open approach. In the same year, the application of an external metallic circle instead of intra-anastomotic stent in low caliber anastomoses in the setting of RYHJ was suggested [30].

Three years later, in the clinical setting, a robotically assisted complete excision of choledochal cyst type I and concomitant extracorporeal RYHJ was performed [31]. The approach was compared with current literature standards on the treatment of choledochal cyst type I and was found noninferior compared to laparoscopic setup. In 2012, the first single-incision laparoscopic RYHJ was performed using conventional instruments in children with choledochal cysts offering noninferior postoperative results in terms of length of hospital stay and time to feed compared to conventional laparoscopic approach [32].

Nowadays, the intermediate-term outcome for totally laparoscopic choledochal cyst excision and RYHJ at a single center in a 5-year period was published and concluded that this procedure is a safe and efficacious procedure for the most instances of adult choledochal cyst demanding advanced laparoscopic skills, good team cooperation, and stapler anastomosis [33].

Table 1 summarizes the crucial milestones of the evolution of the biliary diversion operations throughout the years.

## 3. Our RYHJ Technique

Herein, we describe a step-by-step analysis of our technique as we use it during the last 25 years in more than 400 patients. After careful dissection and division of the extrahepatic, hilar, or intrahepatic bile duct(s) (depends on the operation indication), the arterial blood supply of the proximal cutting edge is checked. In cases of insufficient bleeding from the bile duct stump(s), the preparation is continued cranially, until satisfactory arterial bleeding is observed. Neighboring bile ducts with a small orifice diameter were transformed into a common channel, using one to two PDS 5-0 or 6-0 (PDS®; Ethicon, Hamburg, Germany) interrupted stitches. In order to achieve sufficient bile duct caliber we prefer to open up the left hepatic duct but keeping the posterior wall of the bifurcation, according to the Hepp-Couinaud technique [34]. In case that the stenosis is covered by liver tissue at the liver hilum, the liver tissue has to be removed using ultrasonic dissection (MISONIX, USA). When there is concomitant vascular injury to the hepatic hilum, we try to avoid early reconstruction after the injury, in order to allow arterial supply regeneration. Stay sutures are placed at the anterior surface and at the 2 corners (3 and 9 hours) in order to improve lumen visibility.

The Roux-en-Y jejunal limb is then prepared by transecting the jejunum around 20–30 cm distal from the Treitz ligament. The stapler-line of the Roux-limb is reinforced with interrupted PDS 4-0 sutures and then brought in a retrocolic (anteduodenal, in the cases where the duodenum is present) fashion, right of the middle colic vessels, to the right upper abdomen. Care must be taken to ensure a tension-free jejunal limb with sufficient length.

**Figure 1:**
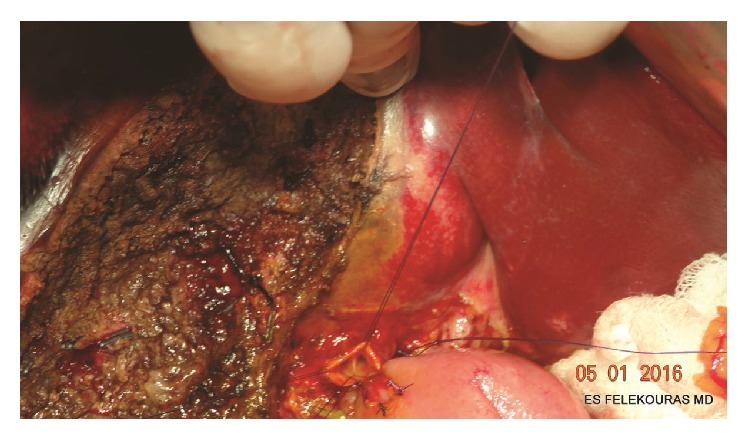
Dissection and division of the extrahepatic bile duct to the level of the biliary confluence of the right and left hepatic ducts at the hilum. Stay sutures with atraumatic needle placed in the stumps of each hepatic duct. The mucosa of the jejunal limb orifice is slightly inverted, using four PDS 5-0 interrupted sutures. Note that the circumference of the duct is 2*πr* = 21.997, so the bites have to be 2*πr*/4 = 21.997/4 = 5,4 mm. So this anastomosis can be done with 6 stiches (assuming you put your stiches with a step of 4 mm).

A small orifice (5 mm) at the antimesenteric side of the Roux-limb and 2-3 cm distal to stapled jejunal stump is created. When a pancreatoduodenectomy is performed, we prefer to leave a distance of 8–10 cm between the pancreaticojejunostomy and the hepaticojejunostomy. The diameter of the jejunal orifice should always be much smaller than the width of the hepatic duct. The mucosa of the intestinal orifice is slightly inverted, using four PDS 5-0 interrupted stitches in a “crosswise” fashion, in order to create a mucosa-to-mucosa anastomosis (Figure 1). The reason for this step is to ensure a well-adapted duct-to-mucosa HJ.

For the construction of our single-layer, end-to-side HJ we use 4-0 to 6-0 PDS interrupted sutures. The first two sutures are placed in the left corner of the jejunum and the bile duct. The needles are passed through the bile duct from outward to the inside and then through the jejunum from the inside outwards. The jejunal limb is then gently pushed down to the hepatic duct and the sutures are tied. All the passings of the stiches take a good amount of seromuscular part of the small bowel* but not* the mucosa, helping the mucosa to be inside the bile duct and so to complete a mucosa-to-mucosa anastomosis. Besides, we have to mention that every bite to the bile duct has to take a good tissue amount, which has to be at least 4-5 mm in order to avoid tearing and ischemia. The number of stiches we use is related to the caliber of the bile duct and long experience showed us that every step to next stich has to be also 4-5 mm. This number comes mainly from experience but can be also helpful to calculate the number of stiches or bites you are going to use for the whole anastomosis based on the perimeter of the duct (Figure 1).

**Figure 2:**
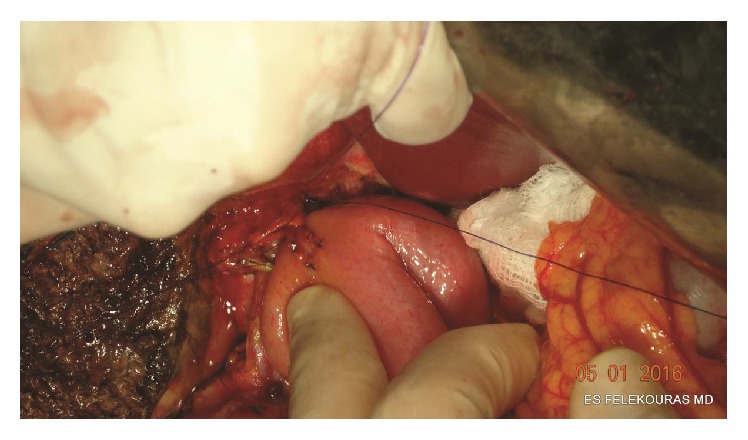
Construction of the posterior wall of the anastomosis. The jejunal limb is gently pushed down to the hepatic duct and the sutures are tied with the knots lying on the outside of the anastomosis.

The posterior wall of the anastomosis is completed by placing the appropriate number of sutures in the same way from the left to the right. All knots of the posterior wall remain outside of the anastomosis (Figure 2). Attention has to be paid not to tear the bile duct during the ligation. In selected cases, where the bile duct diameter and its wall thickness are big enough, this step can also be done in a “running” fashion.

**Figure 3:**
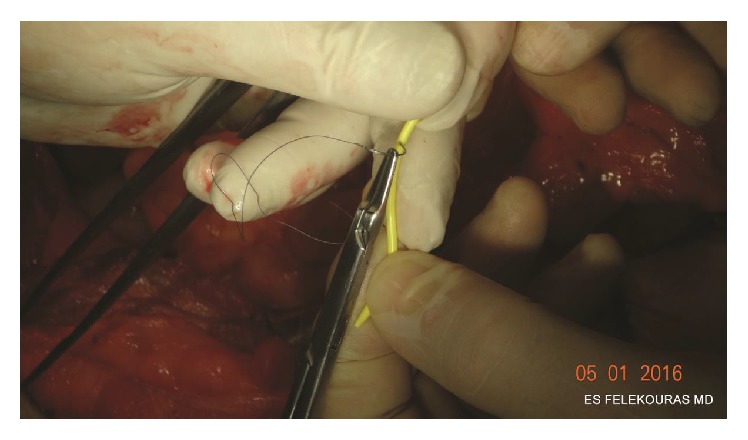
Fixation of the pigtail catheter to the jejunal stump using a 5-0 Vicryl suture.

At this point, we prefer to place a transanastomotic (in-in) stent in order to protect and improve the patency of the anastomosis in the early postoperative period. We usually use an 8-10 French Nelaton catheter or the edge of a 6 Fr. “pigtail” catheter in cases of small bile ducts. The stent is temporarily fixed in place using a 5-0 Vicryl suture (Figure 3). When a PTBD is placed preoperatively, the drainage is preserved and placed intraluminally as an external-internal stent.

**Figure 4:**
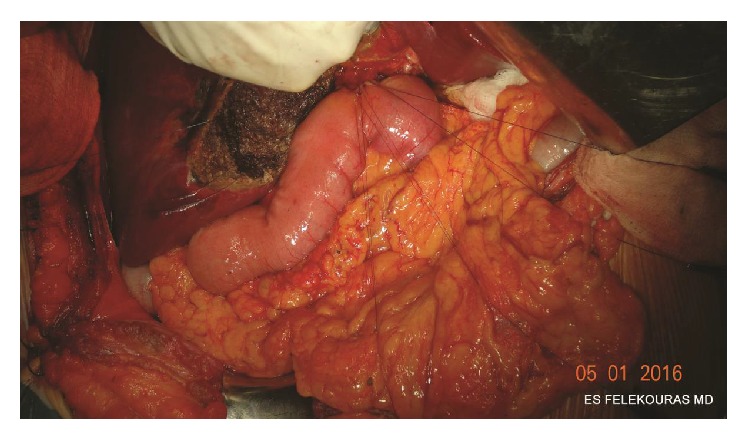
Anterior row of sutures placed to complete the approximation of the jejunum and bile duct.

The anterior wall of the anastomosis is constructed in the same fashion. Suturing should start from the left to the right side, passing the needle through the jejunum outside-inwards and then through the bile duct from the inside to outward. The sutures are then tied, while the inverted mucosa of the jejunum should be buried intraluminally (Figure 4). A small trick to achieve that is to bring the knot of the tie on the bowel site.

**Figure 5:**
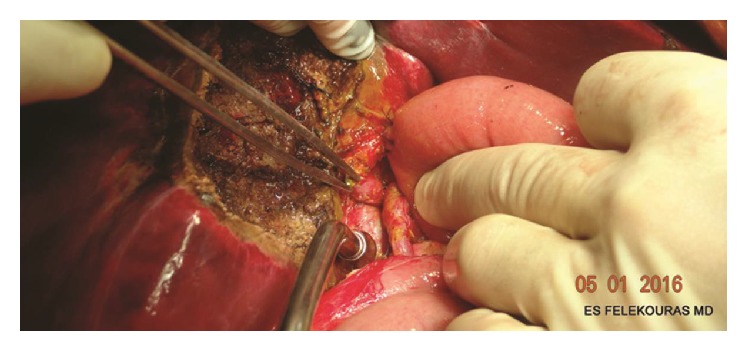
Completion of the hepaticojejunostomy.

After the completion of the anastomosis, control for bile leaks (if present) should be performed (Figure 5). When a PTBD is in place, a “white-test” with propofol or lipiodol can be made in order to check the patency and the integrity of the anastomosis. The mean operative time of the technique is 74 minutes.

We strongly believe that the key-points to the long-lasting results of this technique are the prevention of ischemia, the avoidance of bile leak, and the mucosa-to-mucosa anastomosis.

Despite not being the purpose of our analysis, we will briefly report the outcomes of the application of this technique from 1992 till 2015. During this period, 412 patients underwent biliary diversion with the technique described above. The majority of cases were due to pancreatic or ampullary cancer (29%). Around 25% of the cases were BDIs and 12% of the cases were cholangiocarcinomas. Benign biliary (choledochal cyst, choledocholithiasis, etc.) and pancreatic diseases (chronic and autoimmune pancreatitis) reached almost 22% of the cases. Finally, 50 cases (12%) were performed in liver transplantation setting. The number of anastomotic leaks was 8 (2.1%) and the cases of anastomotic strictures reached 12 (3.1%). Other complications included wound infection (38-10%), biloma (9-2.3%), recurrent cholangitis (11-2.88%), biliary peritonitis (2-0.5%), and others (pulmonary embolism, urinary tract infection, pneumonia, etc./13 cases; 3.4%). The overall morbidity rate was 28.2%. The mortality rate reached 3.9% (15 cases). The majority (12/15-80%) of these patients underwent RYHJ in emergency setting.

## 4. Discussion

The creation of a secure HJ is an essential skill for any hepatobiliary surgeon. And if we take into consideration that an imperfect anastomosis or its failure may lead to reoperations or reinterventions in a patient with recurrent devastating symptoms, the need for well-performed HJ is imperative.

To date, many techniques and approaches have been described. Recently Sutherland and Dixon [35] described a refined technique of sewing the end of the common hepatic duct to the side of the jejunum. The sutures are placed to include all layers of the bowel wall except mucosa. This extramucosal HJ was performed in 185 cases with 1.7% leak rate, a stricture rate of 4.9%, and no mortality [35].

Laukkarinen et al. [36] demonstrated a RYHJ with a transanastomotic biodegradable stent with low rates of anastomotic leakage or stricture in experimental models. The presence of a stent seems to increase the caliber of the anastomosis since postoperative duct diameter was found larger than the preoperative one [36]. Long-term clinical studies are required to confirm these initial experimental findings.

One of the long-standing tenets when performing biliary reconstruction is the use a long hepatic limb to decrease the risk for postoperative cholangitis. Most authors recommend Roux-limbs of up to 75 cm; Felder et al. [37] have routinely used a Roux length of 20 cm to facilitate possible postoperative endoscopic access. In their series they presented less than 6% of anastomotic stricture and 10% of long-term and 3% of immediate complications; most of them required reoperation [37]. We must highlight that almost half of the cases in the series were liver transplantation cases.

Emerging data come from the evaluation of minimal invasive approaches to perform HJ, even in severe BDI injuries. In the laparoscopic setting, it was recently showed that laparoscopic approach to BDI repair is feasible and safe with low morbidity rates (bile leak, 17.2%, reintervention, 6.8%) accompanied with the well-established advantages of laparoscopic surgery (low pain, earlier mobilization, and cosmesis) [38]. In the setting of malignancies, the results are not that satisfactory since the morbidity rate reached 33.3% and mortality was 2.08% in a series of laparoscopic HJ cases for palliative treatment of pancreatic head malignancy [39]. The disadvantages of these series were the short follow-up and the inadequate number of cases. Moreover, it was recently published that E2 BDI injury was successfully treated with robotic-assisted RYHJ [40]. Despite the satisfactory results, this approach is still in its infancy with several disadvantages including the bulky hardware which makes it impossible for the robot to be moved to other theatres, the high learning curve, and the high operating and maintenance cost making it a “forbidden fruit” in the era of financial crisis. The comparison among different technical modalities of performing RYHJ is not always feasible since the indications, the selection of patients, and the surgical experience differ among studies. And although the technique can be meticulously followed, the experience of the surgeon involved in the performance of the anastomosis is the most important issue.

In our institution, we follow a strict evaluation algorithm to each patient referred to us with biliary disease. The postoperative outcomes of our technique are evaluated as categories of wound infection, bile leak, biloma, and biliary peritonitis. The long-term postoperative complications were evaluated as categories of stricture, recurrent cholangitis, defined as the occurrence of two episodes of cholangitis, the need for nonsurgical intervention/dilation (percutaneous drainage of biloma, ERCP and sphincterotomy, and dilation of anastomosis), and the need for reoperation. As we have previously demonstrated, the long-term postoperative morbidity rate of our technique in BDI cases is 26.8% with half of these cases presenting with stricture of the anastomosis with no difference between the early and late intervention group. No patient required reoperation for BDI-related HJ [41]. This rate of anastomotic stricture is noninferior compared to current literature standards and seems rather attractive if one takes into consideration selection bias secondary to the referral pattern [42–44].

Far beyond the surgical stress that an open surgical intervention releases, HJ itself seems to cause many interesting pathophysiological changes. In an animal model it was well described that HJ was associated with less weight gain and colonization of the bile duct with aerobic bacteria,* Escherichia coli*, dominating with concomitant fibrous periportal infiltration [45]. These changes are of potential clinical importance since many of the postoperative complications could be explained to the bactibilia that might be an important factor in the pathogenesis of cholangitis, gallstone formation, and gallstone pancreatitis.

Long-term outcomes in biliary reconstruction are mainly influenced by the level of injury, presence of local inflammation, timing of final repair, type of reconstruction, and experience and expertise of surgeon in these operations and previous attempts of repair in the same or in other institutions. Patients without history of previous interventions, lack of inflammation, lack of complete transection of common bile duct, and greater diameter of bile duct present better operative results, decreased rates of morbidity and mortality, and lower rates of postoperative complications [46, 47].

It is widely accepted that the best results in biliary reconstruction can be achieved in specialized hepatobiliary centers [48]. Nevertheless, many general surgeons without previous experience attempt to repair these injuries, often without proper understanding or characterization of the biliary injury. This may be associated with inferior short-term and long-term outcomes, substantial morbidity, and higher rates of complications [49]. Every failed attempt at repair leads to a decreased bile duct length, making definitive reconstruction more difficult.

## 5. Conclusions

The RYHJ is a reliable and efficient technique of biliary diversion in most cases of biliary obstruction. It has been significantly developed in the last 100 years till our current era of minimally invasive surgery. We analyze by step-by-step approach the RYHJ technique that we perform in our center. It is established to be a feasible approach, with short learning curve, low anastomotic stricture rates, and almost zero anastomotic leakage cases. It can be applied in a variety of diseases and conditions.

## Figures and Tables

**Table 1:** Milestones of the evolution of biliary diversion techniques.

Author	Year	Technique	Novelty
Reid [10]	1921	CJ	First report of the technique
Sanders [12]	1949	HJ	First report of the technique
Allbritten Jr. [15]	1953	RYCJ	First report of the technique
Allen [16] and Warren [17]	1956	CJ	Introduction of a modified CJ technique
Bismuth et al. [18]	1978	RYHJ	Feasibility and safety study
Bismuth et al. [22]	1987	RYHJ	First application in liver transplantation
Röthlin et al. [7]	1998	Lap RYHJ	First retrospective analysis on the safety and feasibility
Nagino et al. [28]	2002	RYHJ	Limb placed via the retrocolic-retrogastric route in obese patients
Kang et al. [31]	2007	Robotic-assisted RYHJ	First experience in clinical setting
Diao et al. [32]	2012	SILS RYHJ	Performed using conventional instruments in children with choledochal cysts
